# Correction: Sałasińska et al. Burning Behaviour of Rigid Polyurethane Foams with Histidine and Modified Graphene Oxide. *Materials* 2021, *14*, 1184

**DOI:** 10.3390/ma15031011

**Published:** 2022-01-28

**Authors:** Kamila Sałasińska, Milena Leszczyńska, Maciej Celiński, Paweł Kozikowski, Krystian Kowiorski, Ludwika Lipińska

**Affiliations:** 1 Department of Chemical, Biological and Aerosol Hazards, Central Institute for Labour Protection–National Research Institute, 00-701 Warsaw, Poland; maciej.celinski@ciop.pl (M.C.); pawel.kozikowski@ciop.pl (P.K.); 2 Faculty of Materials Science and Engineering, Warsaw University of Technology, 02-507 Warsaw, Poland; milena.leszczynska.dokt@pw.edu.pl; 3 Department of Chemical Synthesis and Flake Graphene, Łukasiewicz Research Network–Institute of Microelectronics and Photonics, 01-919 Warsaw, Poland; krystian.kowiorski@imif.lukasiewicz.gov.pl (K.K.); Ludwika.Lipinska@imif.lukasiewicz.gov.pl (L.L.)

In the original article [1], there was a mistake in Table 8 as published. The values from *Table 7* have been inserted twice. The corrected Table 8 *Smoke emission of unmodified PU and polyurethane foams with APP or HGOA system* appears below.

There was an error in the Section 3.5 of the original article. The wrong name of the sample was used (PU/20HGO_A_).

A correction has been made to Results, Fire Behavior, *Section 3.5*:

Since the PU foams are characterized by cellular structure and low thermal inertia [39,40], the time to ignition (TT) of all samples was 6 s or less. Reduced thermal inertia of foams with lower density may lead to decreased TTI values [38], as in the case of PU/10HGO_A_; however, no trend was observed. PHRR were similar within APP and HGO_A_ series independently of FR amount; nevertheless, insignificantly lower values were obtained for PU with HGO_A_. The differences between the pHRR for PU with 10%, 20%, and 30% of APP and HGO_A_ were 21%, 24%, and 21%, respectively. A similar trend was observed for MARHE, and the lowest results were recorded for samples with the lowest amount of additives.

The authors apologize for any inconvenience caused and state that the scientific conclusions are unaffected. The original article has been updated.

## Figures and Tables

**Table 8:** Smoke emission of unmodified PU and polyurethane foams with APP or HGO_A_ system.

Samples	TSR, m^2^/m^2^	SEA, m^2^/kg	Ds_max_	VOF4
PU/10APP	586 (72)	622 (60 ^a^)	142 (10)	308 (15)
PU/20APP	685 (134)	739 (16)	158 (29)	369 (82)
PU/30APP	706 (118)	757 (52)	180 (7)	431 (4)
PU/10HGO_A_	388 (18)	466 (12)	108 (6)	233 (5)
PU/20HGO_A_	298 (13)	359 (79)	127 (18)	226 (2)
PU/30HGO_A_	295 (39)	381 (27)	176 (24)	222 (6)

^a^ The values in parentheses are the standard deviations.
